# Supplementary material for: Altered microRNA Transcriptome in Cultured Human Liver Cells upon Infection with Ebola Virus
Source: Int J Mol Sci. 2021 Apr 6;22(7):3792. doi: 10.3390/ijms22073792 (PMC8038836; doi:10.3390/ijms22073792)
Supplement: Supplementary file 1 [file ijms-22-03792-s001.zip › Supplementary_File/C_ GO_Analysis_Results/16-30nt_go_Reston-24h-Huh7_vs_Control-24h-Huh7_up.mature_mirna_targets/MF_GeneRatioDotPlot.pdf]

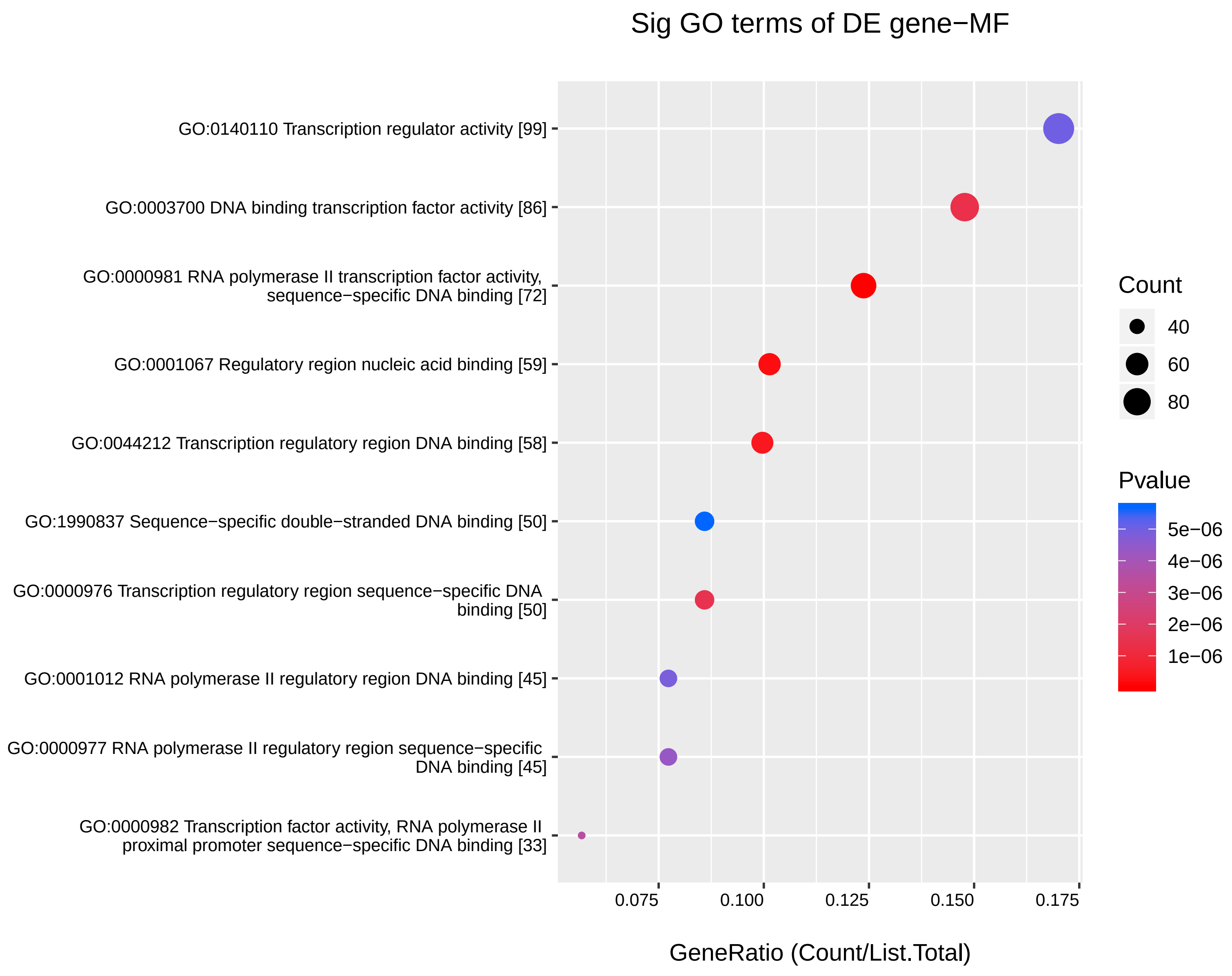

Sig GO terms of DE gene−MF
GO:0140110 Transcription regulator activity [99]
GO:0003700 DNA binding transcription factor activity [86]
GO:0000981 RNA polymerase II transcription factor activity, sequence−specific DNA binding [72]
GO:0001067 Regulatory region nucleic acid binding [59]
GO:0044212 Transcription regulatory region DNA binding [58]
GO:1990837 Sequence−specific double−stranded DNA binding [50]
GO:0000976 Transcription regulatory region sequence−specific DNA binding [50]
GO:0001012 RNA polymerase II regulatory region DNA binding [45]
GO:0000977 RNA polymerase II regulatory region sequence−specific DNA binding [45]
GO:0000982 Transcription factor activity, RNA polymerase II proximal promoter sequence−specific DNA binding [33]
0.075
0.100
0.125
0.150
0.175
GeneRatio (Count/List.Total)
Count
40
60
80
Pvalue
5e−06
4e−06
3e−06
2e−06
1e−06
